# The role of nutrition in children's neurocognitive development, from pregnancy through childhood

**DOI:** 10.3389/fnhum.2013.00097

**Published:** 2013-03-26

**Authors:** Anett Nyaradi, Jianghong Li, Siobhan Hickling, Jonathan Foster, Wendy H. Oddy

**Affiliations:** ^1^Centre for Child Health Research, Telethon Institute for Child Health Research, The University of Western AustraliaPerth, WA, Australia; ^2^School of Population Health, The University of Western AustraliaPerth, WA, Australia; ^3^Centre for Population Health Research, Curtin Health Innovation Research Institute, Curtin UniversityPerth, WA, Australia; ^4^Social Science Research CenterBerlin, Germany; ^5^School of Psychology and Speech Pathology, Curtin UniversityPerth, WA, Australia; ^6^Neurosciences Unit, Health Department of Western AustraliaPerth, WA, Australia; ^7^School of Paediatrics and Child Health, The University of Western AustraliaPerth, WA, Australia

**Keywords:** nutrition, cognitive development, children, micronutrients, diet quality

## Abstract

This review examines the current evidence for a possible connection between nutritional intake (including micronutrients and whole diet) and neurocognitive development in childhood. Earlier studies which have investigated the association between nutrition and cognitive development have focused on individual micronutrients, including omega-3 fatty acids, vitamin B12, folic acid, choline, iron, iodine, and zinc, and single aspects of diet. The research evidence from observational studies suggests that micronutrients may play an important role in the cognitive development of children. However, the results of intervention trials utilizing single micronutrients are inconclusive. More generally, there is evidence that malnutrition can impair cognitive development, whilst breastfeeding appears to be beneficial for cognition. Eating breakfast is also beneficial for cognition. In contrast, there is currently inconclusive evidence regarding the association between obesity and cognition. Since individuals consume combinations of foods, more recently researchers have become interested in the cognitive impact of diet as a composite measure. Only a few studies to date have investigated the associations between dietary patterns and cognitive development. In future research, more well designed intervention trials are needed, with special consideration given to the interactive effects of nutrients.

## Introduction

Cognition represents a complex set of higher mental functions subserved by the brain, and includes attention, memory, thinking, learning, and perception (Bhatnagar and Taneja, 2001). Cognitive development in pre-schoolers is predictive of later school achievement (Tramontana et al., 1988; Clark et al., 2010; Engle, 2010). As Ross and Mirowsky (1999) state: “Schooling builds human capital - skills, abilities, and resources—which ultimately shapes health and well-being.” Indeed, more education has been linked to better jobs, higher income, higher socio-economic status, better health care access and housing, better lifestyle, nutrition, and physical activity (Florence et al., 2008), which are all well-known health determinants. Education increases an individual's sense of personal control and self-esteem; these factors have also been shown to influence better health behavior (Ross and Mirowsky, 1999; Logi Kristjánsson et al., 2010). Academic achievement is important for future personal health, and is therefore a significant concern for public health.

Cognitive development is influenced by many factors, including nutrition. There is an increasing body of literature that suggests a connection between improved nutrition and optimal brain function. Nutrients provide building blocks that play a critical role in cell proliferation, DNA synthesis, neurotransmitter and hormone metabolism, and are important constituents of enzyme systems in the brain (Bhatnagar and Taneja, 2001; Lozoff and Georgieff, 2006; Zeisel, 2009; De Souza et al., 2011; Zimmermann, 2011). Brain development is faster in the early years of life compared to the rest of the body (Benton, 2010a), which may make it more vulnerable to dietary deficiencies.

In this literature review, we assess the current research evidence for a link between nutritional intake in pregnancy and childhood and children's cognitive development. We first discuss individual micronutrients and single aspects of diet, which represents earlier research in this area. We next consider the more encompassing aspects of diet, which have emerged as researchers became more interested in diet as a comprehensive measurement. The most recent research trend in this area suggests a broader analysis of the role of nutrition in neurocognitive development, which we offer here in comparison to previous reviews (Black, 2003b; Bellisle, 2004; Stevenson, 2006; Georgieff, 2007; Benton, 2010a).

## Brain development in humans

The understanding of the functional and structural development of the human brain has emerged from a range of methodologies (including clinical lesion and experimental animal studies) and lately as a result of greatly improved neuroimaging methods, in particular Positron Emission Tomography and Magnetic Resonance Imaging (MRI) (Levitt, 2003; Uddin et al., 2010). Brain development is a temporally extended and complex process, with different parts and functions of the brain developing at different times (Grossman et al., 2003). By 5 weeks after conception in humans, the anterior-posterior and dorsal-ventral axes of the neural tube have already developed (Levitt, 2003). The cortical plate (which is the forerunner of the cerebral cortex) and some inter-neuronal connections form from 8 to 16 weeks of gestation (Kostović et al., 2002; Levitt, 2003). From 24 weeks of gestation until the perinatal period, the neurons in the cortical plate die and are replaced by more mature cortical neurons. During this time, significant refinement in neural connections take place (Levitt, 2003). From 34 weeks post-conception until 2 years of age, peak synapse development, and significant brain growth occurs (Huttenlocher and Dabholkar, 1997; Levitt, 2003). By preschool age, synaptic density has reached the adult level. The myelination of some parts of the brain (particularly those that control higher cognitive functions, such as the frontal lobes) continues well into adolescence, whilst myelination occurs earlier in other parts of the brain that coordinate more primary functions (Toga et al., 2006). Although the gray matter (which contains the bodies of nerve cells) reaches asymptote by the age of 7–11 in different regions of the brain, it is thought that the growth of the white matter (which represents axonal nerve tracts) continues beyond 20 years of age. Studies have shown that the maturation of specific brain areas during childhood is associated with development of specific cognitive functions such as language, reading, and memory (Nagy et al., 2004; Deutsch et al., 2005; Giedd et al., 2010). The development of the frontal lobes, which are believed to control higher cognitive functions (including planning, sequencing and self-regulation), appears to occur in growth spurts during the first 2 years of life, and then again between 7 and 9 years of age and also around 15 years of age (Thatcher, 1991; Bryan et al., 2004). The development of some subcortical structures including the basal ganglia, amygdala, and hippocampus (which are also centrally involved in some mediating higher cognitive functions, including memory, executive functions, and emotion) also continues until late adolescence. In addition, a meta-analysis has confirmed a connection between the size of the hippocampus and memory performance during brain development in children and young adults (Van Petten, 2004). Overall, the research evidence suggests that cognitive development is strongly connected with micro and macro-anatomical changes which take place throughout childhood (Levitt, 2003; Herlenius and Lagercrantz, 2004; Ghosh et al., 2010).

Individual brain development follows a genetic program which is influenced by environmental factors including nutrition (Bryan et al., 2004; Toga et al., 2006; Giedd et al., 2010). Environmental influences may modify gene expression through epigenetic mechanisms, whereby gene function is altered through the processes of DNA methylation, histone modification and the modulating effect of non-coding RNAs, without the alteration of the gene sequence *per se*. These epigenetic factors can cause long lasting or even heritable changes in biological programs (Levi and Sanderson, 2004; Rosales et al., 2009; Murgatroyd and Spengler, 2011; Lillycrop and Burdge, 2012). It has been shown in animal and more recently in human studies that nutrition is one of the most salient environmental factors, and that nutrition can have a direct effect on gene expression (Levi and Sanderson, 2004; Rosales et al., 2009; Attig et al., 2010; Lillycrop and Burdge, 2011; Jiménez-Chillarón et al., 2012). One of the first and best known human studies in the rapidly growing field of “Nutritional Epigenomics” relates to the Dutch Hunger Winter during the 1940's in which the offspring of mothers exposed to famine during pregnancy had an increased risk of cardiovascular, kidney, lung, and metabolic disorders and reduced cognitive functions (Roseboom et al., 2006; De Rooij et al., 2010). More specifically, evidence has been obtained of hypo- and hyper-methylated DNA segments from the blood cells of the affected individuals (Heijmans et al., 2008).

Evidence suggests that the timing of nutritional deficiencies can significantly affect brain development. For example, it is well known that folic acid deficiency between 21 and 28 days after conception (when the neural tube closes) predisposes the foetus to a congenital malformation, called a neural tube defect. Hence, this is a critical period, because during that time an irreversible change in the brain structure and function occurs if there is inadequate folic acid present (Blencowe et al., 2010). A critical period is a specific period within a sensitive timeframe (Knudsen, 2004). A sensitive period tends to reflect a broader timeframe; during such a developmental period the brain is more sensitive to specific interventions. However, skills and abilities can still be acquired outside this time period, albeit with less proficiency (Knudsen, 2004). An example is that deaf children who receive cochlear implants within a sensitive period for brain development (i.e., before the age of 3–5 years) show better language development than those who receive a cochlear implant after this period (Penhune, 2011).

Since rapid brain growth occurs during the first 2 years of life (and by the age of 2 the brain reaches 80% of its adult weight), this period of life may be particularly sensitive to deficiencies in diet (Bryan et al., 2004; Lenroot and Giedd, 2006). Adolescence is also a significant and sensitive developmental period, with research indicating that structural reorganization, brain and cognitive maturation and—in particular—major developments in the prefrontal cortex take place during puberty (Luna and Sweeney, 2001; Sisk, 2004; Peper et al., 2009; Asato et al., 2010; Blakemore et al., 2010).

## Dietary influences on cognitive development

### Micronutrients and cognitive development

#### Omega-3 fatty acids

In recent years, there has been an increasing interest in the effect of essential fatty acids, particularly long chain polyunsaturated fatty acids (LCPUFA), on cognitive brain development. Of the human brain's dry weight 60% is comprised of lipids, of which 20% are docosahexaenoic acid (DHA; which is an omega-3 fatty acid) and arachidonic acid (AA; an omega-6 fatty acid). These represent the two core fatty acids found in gray matter (Benton, 2010b; De Souza et al., 2011). Furthermore, the supply of LCPUFAs from food, especially the omega-3 fatty acids, including DHA and eicosapentaenoic acid (EPA), is frequently inadequate for children as well as for adults (Schuchardt et al., 2010).

Essential fatty acids play a central functional role in brain tissue. They are not only the basic components of neuronal membranes, but they modulate membrane fluidity and volume and thereby influence receptor and enzyme activities in addition to affecting ion channels. Essential fatty acids are also precursors for active mediators that play a key role in inflammation and immune reaction. They promote neuronal and dendritic spine growth and synaptic membrane synthesis, and hence influence signal processing, and neural transmission. In addition, essential fatty acids regulate gene expression in the brain (McCann and Ames, 2005; Eilander et al., 2007; Innis, 2007; Cetina, 2008; Wurtman, 2008; Ramakrishnan et al., 2009; Ryan et al., 2010; Schuchardt et al., 2010; De Souza et al., 2011). Therefore, the existing literature strongly suggests that essential fatty acids are critical for brain development and function.

It has been suggested that the fast growth of the human cerebral cortex during the last two million years was strongly related to the balanced dietary intake of LCPUFAs (Broadhurst et al., 1998), specifically with an equal ratio of omega-6 and omega-3 fatty acids in the diet (Simopoulos, 1999). Evidence proposes that the modern Homo sapiens, whose brain developed significantly relative to its ancestors, lived near rivers and oceans, where seafood and fish were abundant (Crawford et al., 1999). The rise in intellectual and brain development in Homo Sapiens also coincided with tool making and language development (Crawford et al., 1999; Broadhurst et al., 2002). During the last 150 years, it is believed that the balance of omega-6 to omega-3 fatty acids has shifted in favor of omega-6 fatty acids in the diet, resulting in a ratio of 20–25:1 and a dietary deficiency in omega-3 fatty acids (Simopoulos, 1999). A diet that is deficient in omega-3 fatty acids may have health and developmental implications (Simopoulos, 2008).

A number of epidemiological studies have shown a positive association between maternal fish intake (which is a rich source of omega-3 fatty acids) during pregnancy and cognitive development in children (Daniels et al., 2004; Hibbeln et al., 2007; Jacobson et al., 2008; Oken et al., 2008a,b; Boucher et al., 2011). Data from the Avon Longitudinal Study of Parents and Children (ALSPAC) in the UK regarding fish consumption and child cognitive development were analyzed in two studies (Daniels et al., 2004; Hibbeln et al., 2007). The earlier study found evidence that higher maternal fish consumption was associated with higher language and social skills (after appropriate adjustments) in 7421 British children assessed at 15 months, using the MacArthur Communicative Development Inventory (MCDI), and at 18 months using the Denver Developmental Screening test (Daniels et al., 2004). The later ALSPAC study demonstrated that those children whose mothers consumed lower levels of seafood during pregnancy had lower IQ, measured by the Wechsler Intelligence Scale for Children III (WISC-III) at the age of 8 (after adjusting for a wide range of relevant covariates). Lower maternal seafood consumption was also linked to suboptimal behavior at age seven (measured using the Child Behavior Checklist) and to lower levels of social, fine motor and language development (measured using the Denver Developmental Screening test) at six, 18, 30, and 42 months of age in the same study (Hibbeln et al., 2007). Although higher fish intake may result in higher erythrocyte mercury concentration (which has been shown to alter neurodevelopment adversely), research in American schoolchildren (Project Viva, a prospective pre-birth cohort study) demonstrated that higher maternal fish intake was still positively associated with improved language scores on the Peabody Picture Vocabulary Test (PPVT), after adjustment for many potential confounders and covariates (Oken et al., 2008a). The Danish National Birth Cohort study investigated the developmental milestones of 25,446 six- and 18-month old children on a developmental scale created by the researchers, and found that higher maternal fish intake was beneficial for cognitive development even after adjusting for breastfeeding and many sociodemographic factors (Oken et al., 2008b). Two other studies of Inuit children in Arctic Quebec, Canada, showed that higher umbilical cord DHA concentration was associated with: (1) improved infant cognitive development, measured on the Fagan Test of Infant Intelligence at 6 months, (2) on the Bayley Scales of Infant Development test used at 11 months (Jacobson et al., 2008), and (3) better memory performance of school children on both the Digit Span Forward subtest of the WISC-IV and the California Verbal Learning Test-Children Version (Boucher et al., 2011). These results were independent of mercury contamination in seafood. Both studies adjusted for a wide range of socioeconomic and demographic factors. However, these investigations used smaller samples, specifically 109 infants (Jacobson et al., 2008) and 154 schoolchildren (Boucher et al., 2011), compared to the previously described studies. In conclusion, the positive association between maternal fish intake and cognitive development is supported by evidence from the studies cited above.

However, intervention studies of LCPUFA supplementation during pregnancy have produced conflicting results so far. Some studies have reported positive associations between DHA supplementation and cognitive developmental parameters (Helland et al., 2003, 2008; Colombo et al., 2004; Judge et al., 2007; Dunstan et al., 2008). A randomized placebo-controlled double- blind study undertaken by Helland et al. (2003) in Norway used a design which supplemented women from 18 weeks of pregnancy until 3 months postpartum with cod liver oil containing 1183 mg DHA. Children's cognitive status was assessed at 6 and 9 months on the Fagan Test of Infant Intelligence (Helland et al., 2001), and at 4 years of age by the Kaufman Assessment Battery for Children (K-ABC), specifically the Mental Processing Composite comprising the Sequential Processing, Simultaneous Processing, and Non-verbal Scales (which reflects children's problem solving and information processing skills; Helland et al., 2003). The results indicated that maternal DHA supplementation improved children's mental processing skills at 4 years of age (Helland et al., 2003), but not recognition memory at 6 and 9 months of age (Helland et al., 2001). However, in a follow-up study conducted at the age of 7, there was no difference in overall IQ between supplemented versus non-supplemented children, but a positive correlation was observed between the concentration of α-linolenic acid (ALA) and DHA in maternal plasma phospholipid and performance on the Sequential Processing scale (Helland et al., 2008). A possible explanation of these findings is that by 7 years of age cognitive development is influenced by many other intervening factors, and the test battery used in the research may not have been sensitive enough to detect the association between diet and cognition at this later age (Helland et al., 2008). The results of a study reported by Judge et al. (2007) also supported the findings of the previous study, providing evidence that maternal DHA supplementation results in better problem solving ability (speed of processing) in 9 month old infants on the Infant Planning Test, but not on recognition memory evaluated using the Fagan Test of Infant Intelligence. These findings may indicate that DHA is more important in the development of problem solving and processing ability than other cognitive functions such as memory. Visual attention or look duration declines during the first year of life, giving place to more complex mental processing (Frick et al., 1999; Colombo et al., 2004; Judge et al., 2007). Colombo et al. (2004) reported an association between maternal DHA supplementation and faster decline in visual attention during infancy, and better resistance to distractibility during the second year of life. Researchers in Western Australia supplemented a small sample of women (*n* = 98) from 20 weeks of gestation until delivery with high dose (2200 mg) DHA or olive oil and showed significant improvements in hand and eye coordination in the supplemented group at 2½ years of age, after adjusting for maternal age, maternal education, and breastfeeding (Dunstan et al., 2008). These researchers also demonstrated better performance in other elements of cognitive development (locomotor, social, speech and hearing performance, and practical reasoning), evaluated using the Griffiths Mental Development Scales, and on language development, evaluated using the PPVT (Dunstan et al., 2008). However, these latter differences were not statistically significant, perhaps due to the relatively small sample size in the study.

Contrary to expectations, some studies did not find a relationship between LCPUFA supplementation during pregnancy and cognitive development in children (Tofail, 2006; Makrides et al., 2010; Campoy et al., 2011). One of these studies was conducted in a developing country, Bangladesh, where a high proportion of mothers suffer from undernutrition, and possibly from multiple micronutrient deficiencies important for brain development. The pregnant mothers in this study were randomized into fish-oil or soy-oil supplemented groups, and their infants' cognitive development was measured on the Bayley Scales of Infant Development at ten months of age. In this study, the mothers were only given supplements during the third trimester, which may not have represented a sufficient timeframe for supplement administration (Tofail, 2006). In a recent study conducted in Europe on 270 women from three countries, cognitive development was measured on the K-ABC at 6½ years of age after 500 mg DHA prenatal supplementation. In this study, the co-authors did not find significant differences in intelligence between the intervention and control groups (Campoy et al., 2011). The explanation offered was that the positive effect of prenatal supplementation may have been overshadowed by other important factors (not all of which it is possible to control for) including social stimulation, other nutrients taken, diet as a whole, illnesses, and drugs prescribed by the age of 6–7 years (Helland et al., 2008). Makrides et al. (2010) conducted a well-designed multicenter double-blind randomized controlled trial in Australia on 2399 women between 2005 and 2009 from 21 weeks of gestation until birth and did not find any difference on the Bayley Scales of Infant and Toddler Development at the age of 18 months between intervention (supplemented with 800 mg DHA) and control groups (supplemented with vegetable oil capsules), after adjustment for potential confounders. Given the size of this study, the Makrides et al. (2010) investigation was certainly adequately powered to detect a statistically significant difference; yet, no such difference was observed.

Some published studies have also considered supplementation in lactating mothers in order to examine the effect of increased omega fats in breast milk on the cognitive development of children. Reviews of these studies have concluded that there are indications that supplementing lactating mothers with fish oil may positively influence cognitive development in children (Eilander et al., 2007; Hoffman et al., 2009).

In conclusion, the current findings show inconsistencies in the efficacy of maternal supplementation with omega-3 fatty acid. In seeking to account for the contrasting findings, it seems that the following considerations may be relevant: the interventions were applied in different groups of women, using a wide range of DHA dosage, with different durations of supplementation, and the outcomes were measured on different cognitive instruments and at different ages. The more consistent results obtained in epidemiological studies (compared with supplementation trials using only omega-3 fatty acid) may be explained by the possibility that fish is a whole food, and it contains other nutrients important to cognitive development. Furthermore, by eating fish rather than taking fish supplements, other possibly unhealthy or potentially inflammatory foods may also be displaced—i.e., red meat and processed meats. Epidemiological studies may also be better powered but they may also potentially have less control for confounding.

Postnatal studies have considered the effect of omega-3 fatty acid supplementations on term and preterm infants. Epidemiological studies and supplementation trials have also been undertaken in older children in relation to cognitive development. The DHA component is believed to be one of the main reasons why breast milk may improve the cognitive performance of children. Humans are able endogenously to synthesize DHA from precursor α-linolenic acid. However, the conversion rate varies according to genetically determined polymorphisms in two genes, namely FADS1 and FADS2. Moreover, in infants the conversion to DHA seems to be very limited (Hoffman et al., 2009; Guesnet and Alessandri, 2011). Research consistently shows that the blood levels of DHA in formula-fed infants are lower than in breastfed infants, irrespective of the level of precursors in formulas (Hoffman et al., 2009). Therefore, this topic has generated a great deal of scientific interest, especially with respect to the results of clinical trials that have supplemented infant formulas with LCPUFA and investigated the relationship with cognitive development in either term or preterm infants.

A Cochrane review undertaken by Simmer et al. (2008) included 11 studies and concluded that there is currently not enough evidence to support the supplementation of infant formulas with LCPUFA to benefit cognitive development in children born at term. Another review by McCann and Ames (2005) considered animal as well as human supplementation studies. These authors found that, although animal studies provided convincing evidence for DHA supplementation and improved cognitive performance, these studies were undertaken under extreme dietary conditions in which (in most cases) animals were severely deficient in essential fatty acids. By contrast, in formula and breastfed infants the differences in brain DHA concentrations are small, such that the positive effects of LCPUFA on cognitive development may be difficult to detect. Eilander et al. (2007) also concluded that there is no evidence that formula supplementation in term infants is beneficial for cognitive development. On the other hand, Hoffman et al. (2009) reviewed 20 studies and suggested that those trials which supplemented formula milk with a similar level of DHA to breast milk (i.e., to an average of 0.32%) found beneficial outcomes. A major intake of DHA in the brain happens in the last trimester of pregnancy; therefore, preterm infants are disadvantaged and have decreased brain concentration of this vital LCPUFA. Eilander et al. (2007) indicated that supplementing formula milk with LCPUFA may be beneficial for the cognitive development of preterm infants. However, a recent Cochrane review undertaken on preterm infants reported no significant outcome of supplementation with LCPUFA on their cognitive development (Schulzke et al., 2011).

Since brain development continues through childhood, there have been much interest in the association between cognitive development and omega-3 fat levels through diet and/or supplementation in children. Ryan et al. (2010) reviewed the available epidemiological studies and supplementation trials to date and concluded that, although the results were inconsistent, it appears that those studies that supplemented with higher doses and longer durations of DHA achieved a favorable positive outcome in cognitive development in childhood.

#### Vitamin B12, folic acid, and choline

B12 and folate deficiency resulting in anaemia is rare around the world. However, it can occur in both developing and developed countries especially in older people, in those with absorption problems and in vegetarians (De Benoist, 2008). Folate fortification of bread products has been made mandatory in Australia and in many other countries, which has reduced this deficiency significantly (Brown et al., 2011). In recent years, there has been an increasing interest in the association between vitamin B12, folic acid, choline metabolism, and cognitive development. Folate affects neural stem cell proliferation and differentiation, decreases apoptosis, alters DNA biosynthesis, and has an important role in homocysteine and S-adenosylmethionine biosynthesis (Zeisel, 2009; Zhang et al., 2009). It is believed that choline has similar roles in brain development as folate (Meck and Williams, 2003; McCann et al., 2006; Zeisel, 2006a,b, 2009; Signore et al., 2008). Furthermore, folate, choline and vitamin B12 metabolism are interconnected at the homocysteine-methionine-S-adenosylmethionine pathway (Zeisel, 2009). S-adenosylmethionine is one of the main methyl donors in different metabolic methylation reactions, including DNA methylation. Therefore, choline and folate deficiency may result in DNA hypomethylation, thereby altering gene transcription (Zeisel, 2009). In addition, choline is a component of phospholipids in cell membranes and a precursor for the neurotransmitter acetylcholine (Zeisel, 2006b). Vitamin B12 has a role in axon myelination that is important for impulse conduction from cell to cell, and it also protects neurons from degeneration. Vitamin B12 may also alter the synthesis of different cytokines, growth factors and oxidative energy metabolites such as lactic acid (Dror and Allen, 2008).

In children, the association between vitamin B12 and cognitive development has been mainly observed in infants born of vegetarian/vegan mothers or mothers on a macrobiotic diet. These diets can result in vitamin B12 deficiency, as vitamin B12 is largely found in animal products. A pooled analysis that included 48 case studies of infants with vitamin B12 deficiencies reported a variety of abnormal clinical and radiological signs, including: hypotonic muscles, involuntary muscle movements, apathy, cerebral atrophy, and demyelination of nerve cells (Dror and Allen, 2008). After vitamin B12 treatment, a rapid improvement in neurological symptoms is reported in deficient infants, but many of these infants remained seriously delayed in cognitive and language development in the longer term (Dror and Allen, 2008). The long-lasting effect of vitamin B12 deficiency is supported by the findings of Louwman et al. (2000). These researchers investigated the cognitive functioning of adolescents who consumed a macrobiotic diet until the age of 6 years, compared to children with an omnivorous diet. Those adolescents who consumed a macrobiotic diet until 6 years of age had lower levels of fluid intelligence, spatial ability and short term memory (even with currently normal vitamin B12 status) than the control subjects. Although vitamin B12 deficiency is not likely to occur in non-vegetarian people in western countries, Pepper and Black (2011) raised concern about the more frequent gastric bypass surgeries in obese women and the increased incidence of coeliac disease and inflammatory bowel diseases (such as Crohn's and ulcerative colitis). In these conditions, the absorption of vitamin B12 is substantially decreased in the intestine, thereby potentially adversely affecting the development of future children born to these women.

The association between maternal blood folate status and cognitive development has been investigated in several studies (Tamura et al., 2005; Veena et al., 2010). Tamura et al. (2005) did not find any relationship between maternal blood folate status (“low” vs. “normal”) during the second half of the pregnancy and cognitive development of their children at the age of 5–6 years on different cognitive tests including Differential Ability Scales, Visual and Auditory Sequential Memory, Knox Cube, the Gross Motor Scale and the Grooved Pegboard. These researchers conducted their study among African-American women of low socioeconomic status and their disadvantaged children. These children nevertheless did not present with signs or symptoms of any overt clinical deficiency (such as megaloblastic anaemia). On the other hand, Veena et al. (2010) reported that higher maternal blood folate concentration—but not folate status (“low” vs. “normal”, similar to the previous study)—was associated with better cognitive performance on a wide range of tests (Atlantis, Word Order, Pattern Reasoning, Verbal Fluency, Koh's Block Design and WISC-III) in 9–10-year-old Indian children. Interestingly, most of the women (96%) in this study manifested blood folate levels within the normal range. However, the only positive association found between maternal vitamin B12 status and cognitive performance in these children was on verbal fluency, although almost half of the mothers (42.5%) manifested moderately low vitamin B12 level. It is possible that vitamin B12 affects some cognitive functions only if the person is severely deficient, as can be seen in vegetarian mothers and their children. Another reason for the limited findings may be that the tests conducted were not sensitive enough to detect small changes in different functions. Another research group in India showed that lower vitamin B12 levels during pregnancy impaired short term memory (Digit Span Test) and sustained attention (Color Trail Test) in 9 years old children after adjusting for covariates (Bhate, 2008) while non-verbal intelligence on Raven's Cultured Progressive Matrices and visual perception on a Visual Recognition Test were unaffected in this study.

Although there is no sufficient data about the requirements of choline in humans, choline does not seem to be deficient in the general population, with the exception of experimental conditions (Commonwealth of Australia, 2006). Evidence from numerous animal studies indicates that dietary choline has an important impact on the cognitive development of offspring (Meck and Williams, 2003; McCann et al., 2006). Choline in animal models alters the development of the hippocampus, which has a central role in memory and learning (Zeisel, 2006a). Like folate, choline also has a role in the closure of the foetal neural tube. A study among Californian mothers found an increased risk of neural tube defects of their children with lower maternal dietary choline intake, as identified from a food frequency questionnaire (Shaw et al., 2004). There is only one study to date that has evaluated the impact of maternal blood choline (represented across a wide range of concentrations) on intelligence (measured on the Wechsler Preschool and Primary Scale of Intelligence-Revised) in 5 year old children. However, the authors did not find a significant correlation between the two (Signore et al., 2008). In summary, the impact of vitamin B12, folate and choline on children's cognitive development has not been adequately researched to date in humans.

#### Zinc

Zinc deficiency appears to be a major problem worldwide, affecting 40% of the global population (Maret and Sandstead, 2006). Recent research suggests that toddlers, adolescents, older people and individuals with diabetes are possibly at a higher risk of zinc deficiency in Australia (Gibson and Heath, 2011). Animal studies have established a relationship between zinc and neurodevelopment (Shah and Sachdev, 2006; Summers et al., 2008; Coyle et al., 2009). It is believed that zinc is a vital nutrient for the brain, with important structural and functional roles (Bhatnagar and Taneja, 2001; Black, 2003a; Bryan et al., 2004; Shah and Sachdev, 2006; Georgieff, 2007). More specifically, zinc is a cofactor for more than 200 enzymes that regulate diverse metabolic activities in the body including protein, DNA and RNA synthesis. In addition, zinc plays a role in neurogenesis, maturation, and migration of neurons and in synapse formation (Bhatnagar and Taneja, 2001; Black, 2003a; Bryan et al., 2004; Shah and Sachdev, 2006; Georgieff, 2007). Zinc is also found in high concentrations in synaptic vesicles of hippocampal neurons (which are centrally involved in learning and memory), and seems to modulate some neurotransmitters including glutamate and gamma- aminobutyric acid (GABA) receptors (Bhatnagar and Taneja, 2001; Levenson, 2006).

Zinc supplementation has a positive effect on the immune status of infants and may prevent congenital malformations (Shah and Sachdev, 2006). However, the relationship between maternal zinc status and the child's cognitive development has not been fully investigated. In an observational study, low maternal zinc intake in Egyptian mothers was associated with lower levels of focused attention in newborns, measured with the Brazelton Neonatal Behavior Assessment Scale (Kirksey et al., 1994). Surprisingly, a placebo controlled randomized trial undertaken on poor Bangladeshi mothers found that 13 months old infants of zinc supplemented mothers scored less on the Bayley scales of infant development than infants born to mothers who received a placebo (Hamadani et al., 2002). In trying to explain their findings, these researchers argued that zinc supplementation alone may cause imbalances or even deficiencies of other micronutrients that are important for brain development, as micronutrients interact with one another (Hamadani et al., 2002)—a point which we will return to later in this review. Another double-blind randomized controlled trial of maternal zinc supplementation among African-American mothers showed no difference in the cognitive development of 5 years old children between the intervention and control groups, measured on the Differential Ability Scales, Visual and Auditory Sequential Memory, Knox Cube, Gross Motor Scale, and Grooved Pegboard (Tamura et al., 2003). In both studies, the mothers were supplemented only in the second half of their pregnancy. Overall, there are only a limited number of studies on this topic. Taken together, the findings do not consistently show a positive relationship between maternal zinc status and cognitive development of children.

Two articles that reviewed earlier observational and randomized control trials in children on zinc and cognitive development concluded that zinc deficiency can negatively influence cognitive development. Conversely, more recent randomized control trials from India (Taneja et al., 2005) and Bangladesh (Black et al., 2004), where malnutrition is common among children, did not find that zinc supplementation alone affects infants' cognitive development on the Bayley Scales of Infant Development test. Nevertheless, in the Bangladeshi trial, when zinc was combined with iron supplementation, it showed an improvement in cognition (Black et al., 2004). Additional studies are therefore needed to examine the long term benefit of zinc on brain development.

#### Iron

One of the most common nutritional deficiencies in both developing and developed countries is iron deficiency. In some parts of the world, such as in Sub-Saharan Africa and South-East Asia, the prevalence is more than 40%. In developed countries—including Australia—it could be as high as 20%, particularly in pregnant women and in children (World Health Organization, 2008). Over the past decades, a considerable literature has been published on the association between iron status/anaemia and cognitive development in children, as well as in animal models (Grantham-McGregor and Ani, 2001). It is believed that iron is involved with different enzyme systems in the brain, including: the cytochrome c oxidase enzyme system in energy production, tyrosine hydroxylase for dopamine receptor synthesis, delta-9- desaturase for myelination, and fatty acid synthesis, and ribonucleotide reductase for brain growth regulation (Deungria, 2000; Lozoff and Georgieff, 2006; Georgieff, 2007; Rioux et al., 2011). In addition, iron appears to modify developmental processes in hippocampal neurons by altering dendritic growth (Jorgenson et al., 2003; Lozoff and Georgieff, 2006).

There are a limited number of studies that have examined the connection between maternal iron status or maternal iron supplementation and cognitive development. (In the below, treatment refers to anaemic individuals, and supplementation to non-anaemic children.) Tamura et al. (2002) found significantly inferior performance in language skills, fine motor skills and attention (and lower but not significant scores in every other test) in 5 years old children whose cord ferritin levels lay in the lowest quartile. Cognitive performance in this study was measured on the WISC-R, the Test for Auditory Comprehension of Language, fine and gross-motor scales of the Peabody Developmental Motor Scales and the Yale Children's Inventory for attention and tractability. The mothers who took part in the study were of African-American descent and low socioeconomic status, and a high proportion of the children were born small-for-gestational-age. However, a randomized placebo controlled iron supplementation trial in a representative sample of Australian pregnant women failed to find any difference between an iron supplemented vs. placebo group in the IQ of children at 4 years of age on the Stanford–Binet Intelligence Scale, despite maternal iron status having improved due to supplementation (Zhou et al., 2006). The authors suggested that supplementing pregnant women who are generally well-nourished with iron may not confer any additional health benefits, while Tamura's study was undertaken in disadvantaged mothers and small-for-gestational-age infants (Tamura et al., 2002; Zhou et al., 2006). A recent trial in Canada by Rioux et al. (2011) also found no evidence that better maternal iron and DHA status enhanced cognitive development in six months old infants, measured on the Brunet-Lezine Scale of Psychomotor Development of Early Childhood and the Bayley Scales of Infant Development. These researchers recruited a small sample size of mothers from a higher socioeconomic background and with better feeding practices, consistent with the methodology and findings of the Australian study cited above.

In children, the relationship between iron and cognitive development has been well researched. In addition, these investigations have been reviewed many times during the last decade. Grantham-McGregor and Ani (2001) reviewed a range of longitudinal studies and reported that anaemic infants had poorer cognitive and school performance in the long term, and that short-term iron treatment trials in anaemic children did not show benefits in cognitive development. A Cochrane review based on seven randomized controlled trials reached a similar conclusion, i.e., that short term iron treatment for anaemia in children less than 3 years old did not improve cognitive development (Logan et al., 2001). Sachdev et al. (2005) included 17 randomized controlled trials in their meta-analysis, and did not find convincing evidence of an association between iron supplementation and treatment for anaemic and cognitive development. However, treating older children with iron deficiency increased IQ significantly. A more recent review and meta-analysis on children (aged 6 years and older), adolescents and adults found that iron treatment increased IQ in anaemic individuals, but iron supplementation did not improve IQ in non-anaemic children (Falkingham et al., 2010).

In summary, there is a lack of epidemiological evidence or data from well-designed intervention trials demonstrating the impact of maternal iron supplementation on the cognitive development of healthy children. There is evidence that older anaemic children benefit from iron treatments. However, cognitive performance tests including the Bayley Scales of Infant Development and the Denver Developmental Screening Test may not be sensitive enough to detect small changes in short-term supplementation or treatment in young children (Armstrong, 2002). Furthermore, if iron deficiency occurs in very early life, the damage may be irreversible, and it may not be possible to reverse this damage with iron treatment (Beard, 2008).

#### Iodine

Iodine deficiency is a significant worldwide public health issue, especially in children and during pregnancy (World Health Organization, 2004). In Australia, the majority of children and pregnant women are mildly deficient in iodine, with some groups reaching moderate to severe deficiency (Gallego, 2010). Iodine deficiency in the soils in many countries has led to food fortification, most commonly the use of iodized salt (World Health Organization, 2004). The relationship between iodine and cognitive development is extensively researched. It is well known today that severe iodine deficiency during pregnancy may cause “cretinism” in children (Forrest, 2004; Zimmermann, 2007, 2009, 2011; Melse-Boonstra and Jaiswal, 2010). The clinical manifestation of cretinism depends on the severity of iodine deficiency; the features may include mental retardation, speech and hearing impairment, upper motor neuron and extrapyramidal lesions (Delong et al., 1985). Iodine is necessary for the production of thyroid hormones in the body; 70–80% of it is found in the thyroid gland (Melse-Boonstra and Jaiswal, 2010). Iodine deficiency manifests in hypothyroidism, causing underproduction of thyroid hormones including triiodothyronin (T3) and thyroxin (T4). Thyroid hormones play an important role in neurodevelopment and numerous neurological processes including neuronal cell differentiation, maturation and migration, myelination, neurotransmission, and synaptic plasticity (Zimmermann, 2009, 2011; Melse-Boonstra and Jaiswal, 2010). In addition, in animal models hypothyroidism alters neurogenesis and the development and functions of synapses in the hippocampus (Desouza et al., 2005; Gong et al., 2010).

Qian et al. (Qian, 2005) conducted a meta-analysis on studies from different locations in China where the soil is severely iodine deficient, and found a 12.3 point decrease in the IQ of those children whose mothers lived in iodine deficient areas compared to those living in iodine sufficient locations. The association between mild-moderate maternal iodine deficiency and cognitive development is not as clear as it is when iodine deficiency is severe (Forrest, 2004; Zimmermann, 2007, 2009, 2011; Melse-Boonstra and Jaiswal, 2010). In mild-moderate iodine deficiency, maternal thyroid stimulating hormone (TSH) and the thyroid hormone T3 level are unaffected, such that hypothyroidism is not clinically or even sub-clinically diagnosed. In such situations the level of maternal T4 may not be sufficient for the appropriate neurological development of the foetus (Melse-Boonstra and Jaiswal, 2010).

A number of observational studies from iodine sufficient or mildly iodine deficient areas of USA, Russia, The Netherlands, Italy and Spain have shown a significant association between mild maternal thyroid deficiency and cognitive impairment in children. The tests that were reviewed in these studies included the WISC, Neonatal Behavioral Assessment Scale, Bayley Scale of Infant Development, McCarthy Scales of Children's Abilities and the Gnome Mental Development Scale (Haddow et al., 1999; Pop, 2001; Pop et al., 2003; Vermiglio et al., 2004; Riano Galan et al., 2005; Kasatkina et al., 2006; Kooistra et al., 2006). By contrast, one study did not find any relationship between maternal T4 levels and cognitive development in children at 6 months (visual recognition memory) and 3 years of age (PPVT and Wide Range of Visual Motor Ability). However, this study included only a very small number of women with abnormal thyroid function (Oken et al., 2009). Berbel et al. (2009) carried out a trial in Spain that showed better gross and fine motor coordination and socialization (Brunet-Lezine Scale) in 18-month-old children whose mothers were supplemented with iodine from early pregnancy, compared to those who took the supplement from late pregnancy. Velasco et al. (2009) also found that those infants whose mothers took daily iodine supplements from the first trimester of pregnancy exhibited better psychomotor development (measured on the Bayley Scales of Infant Development), compared to those whose mothers were not supplemented with groups evaluated at different ages (5.5 and 12.4 months, respectively). Contrary to expectation, another study from Spain reported lower psychomotor development (measured on the Bayley Scale of Infant Development) in infants (especially in girls) born to mothers with maternal multivitamin supplementation that contained high amounts of iodine (100–149 μg/day), when compared to those infants with lesser amounts of maternal iodine supplementation (<100 μg/day) (Murcia et al., 2011). It is possible that the optimum dose of iodine for those mothers who are manifesting only mild iodine deficiency is less; further research is needed to determine the safe level of iodine intake for mildly deficient pregnant women.

The vast number of studies on the iodine status and supplementation in children and its relationship to cognitive development in mild-moderate iodine deficient areas of the world has been reviewed several times. An earlier review and meta- analysis of 18 studies found a 13.5 point difference in IQ between iodine sufficient and iodine deficient children (Bleichrodt and Born, 1994). Other reviews reported that most observational studies on iodine deficient children found some degree of cognitive impairment (when compared to children from iodine sufficient areas), and iodine supplementation trials in school age children have provided some promising results with respect to improvement of some cognitive processes (Zimmermann, 2007, 2011; Melse-Boonstra and Jaiswal, 2010). More recent iodine supplementation trials from Albania and New Zealand found that supplementation of mildly iodine deficient 10–13 years old children improved matrix reasoning in both studies. In addition, fine motor skills and visual problem solving were improved in the Albanian trial (Zimmermann, 2006; Gordon et al., 2009). Relatively few studies have been conducted in very young children to support the significance of iodine in cognitive development (Melse-Boonstra and Jaiswal, 2010).

In conclusion, the above literature suggests that iodine is important for the cognitive development of older children. Furthermore, although iodine supplementation is critical for severely iodine deficient pregnant women, there is no general consensus about the effectiveness of iodine supplementation during pregnancy in countries with mild iodine deficiency.

#### Multivitamin and mineral supplementation

Although it is important to investigate nutrients individually, deficiencies of nutrients rarely occur in isolation, and an inadequate diet typically causes multiple micronutrient deficiencies. In addition, nutrients interact with each other and do not work separately (Benton, 2010a). Thus, it is important to investigate the association between multiple mineral and vitamins supplementation or deficiencies and cognitive development.

A recent systematic review of prenatal maternal micronutrient supplementation and children's cognitive and psychomotor development considered 18 studies, including six multiple-micronutrient supplementation trials. This review found some evidence that multivitamin and mineral supplementation might positively influence certain aspects of brain development in children (Leung et al., 2011). The review included six trials on multiple-micronutrient supplementations conducted in Peru, rural Taiwan, Tanzania (on HIV infected mothers), and in rural China, Indonesia and Bangladesh, where mothers were poorly nourished (Joos et al., 1983; Schmidt et al., 2004; McGrath et al., 2006; Tofail et al., 2008; Li et al., 2009; Caulfield et al., 2010). A very recent randomized controlled trial in Indonesia found that multiple micronutrient supplementation in undernourished pregnant mothers resulted in improved motor development, visual attention and spatial ability in pre-schoolers (Prado et al., 2012). All the above-mentioned trials are from low income countries, it is currently unknown whether the cognitive development of children of well-nourished mothers from higher income countries would benefit from multiple-micronutrient supplementation.

More consistent results from trials supplementing children with multiple-micronutrients have been shown. A meta-analysis investigated 20 randomized controlled trials published from 1970 to 2008 in developed as well as developing countries, and found that multiple-micronutrient supplementation may result in higher fluid intelligence (Eilander et al., 2010). However, this increase was only marginal, and no association was observed with crystallized intelligence in children. The finding of this review (i.e., that fluid intelligence, but not crystallized intelligence, may be influenced by multiple-micronutrient supplementation) is consistent with conclusions drawn from other studies (Benton, 2001, 2012). Fluid intelligence refers to reasoning ability that reflects the individual's current neurological potential (indexed by measures such as speed of processing) rather than their level of past attainment and acquired, crystallized knowledge (which is measured by abilities such as depth of vocabulary). Fluid ability is typically measured via non-verbal cognitive tests, while crystallized intelligence is more usually measured by administering verbal cognitive tests (Eilander et al., 2010; Benton, 2012).

### Overall diet, food, and cognitive performance

#### Breastfeeding

A considerable amount of literature has been published on the possible connection between breastfeeding and cognitive development. Many of these studies demonstrate significantly positive associations between the two; however, the associations typically diminish or are no longer significant after controlling for confounders including maternal IQ, which is believed to be the strongest predictor of children's intelligence (Rey, 2003; McCann and Ames, 2005; Michaelsen et al., 2009). Furthermore, it remains unclear whether the remaining, diminished associations between breastfeeding and child cognitive development are further confounded by factors that have not been controlled for (Michaelsen et al., 2009). A meta-analysis of 20 studies undertaken in the late 1990's found that breastfeeding in normal birth weight infants increased IQ by 2.7 points and in low birth weight children by 5.2 points, but only six of the studies controlled for maternal IQ (Anderson et al., 1999). Three critical reviews conducted in the early 1990's concluded that the evidence linking breastfeeding and cognitive development has not yet been comprehensively demonstrated (Drane and Logemann, 2000; Jain et al., 2002; Rey, 2003). However, a more recent review by Michaelsen et al. (2009) concluded that the majority of studies found an association between breastfeeding and cognitive development, even after adjusting for confounders, and the difference in IQ related to breastfeeding is around 2–5 points at any age. This finding is supported by a large randomized control trial, where breastfeeding mothers were randomized into a breastfeeding promotion intervention trial that resulted in a higher breastfeeding rate up to 12 months after birth (43.3 vs. 6.4%). Intelligence tests were conducted at age 6½ years on the children in both groups (i.e., intervention vs. control) and associations between longer exclusive breastfeeding and improved cognitive development were found (Kramer et al., 2008). As noted earlier in this review, it has been suggested that one of the reasons behind the advantage of breastfeeding over formula feeding concerns the concentration of LCPUFA in breast milk, especially DHA (Michaelsen et al., 2009).

More recently, some studies have directly examined the effect of breastfeeding on brain development and structure. A study by Kafouri et al. (2012) reported that longer breastfeeding duration is positively associated with cortical thickness in the parietal lobe in adolescents, and in the same study they also found an association between intelligence (measured on WISC) and longer breastfeeding after adjusting for relevant confounders, which included maternal education. Herba et al. (2012) used cranial ultrasound in 2 months old infants; those infants who were breastfed exclusively had larger gangliothalamic diameter and head circumference, and smaller ventricular volume compared to bottle fed babies. Furthermore, breastfeeding has been associated previously with not only higher IQ (measured on the WISC) in adolescents but with increased white matter volume, especially in boys (Isaacs et al., 2010).

In summary, the debate concerning whether breastfeeding and cognitive development have a positive association appears to continue, but with more advanced neuroimaging technologies now available, future research may offer greater insights. Nevertheless, as Gökçay (2010) pointed out, breast milk provides the best nutritional intake for infants, regardless of its putative association with cognitive development.

#### Malnutrition

The number of malnourished (undernourished) children continues to rise in some regions, such as in Sub-Saharan Africa (De Onis, 2000). Every year, 20 million newborns (15.5% of all births) are low birth weight, most of them from developing countries (United Nations Children's Fund and World Health Organization, 2004). The effect of malnutrition on brain structures has been extensively researched in animal models. Malnutrition appears to alter cell numbers, cell migrations, myelinisation, synaptogenesis, hippocampal formation and neurotransmission in rats (Debassio et al., 1996; Mathangi and Namasivayam, 2001; Granados-Rojas et al., 2002; Alamy and Bengelloun, 2012). In a human study, researchers described fewer numbers of neurons with shorter dendrites and abnormal dendritic spines in individuals with malnutrition; however, this study was carried out just on 13 severely undernourished infants, compared to seven adequately fed babies (Benítez-Bribiesca et al., 1999). Because of small sample size, this study cannot provide definitive evidence of the effect of malnutrition on brain structure (although the fact that significant differences were observed even with a relatively small number of participants is potentially revealing, in terms of statistical power considerations). Moreover, malnourished children have less energy and interest for learning that negatively influences cognitive development (Engle, 2010).

Malnutrition can develop *in utero*, when the mother is malnourished (as often happens in low income countries). In Western countries, restricted foetal growth is often the result of a medical condition such as severe hypertension, or if the mother consumes higher levels of alcohol (Henriksen and Clausen, 2002; Feldman et al., 2012; Mustafa et al., 2012). For example, in uncontrolled severe hypertension during pregnancy the placental blood flow is restricted and there are placental abnormalities, which may prevent the foetus from obtaining the required oxygen and nutrients for development (Henriksen and Clausen, 2002). It has been shown that intrauterine growth retardation (IUGR) or small-for-gestational age (SGA) at birth is associated with cognitive developmental delays and a decrease of 4–8 points in IQ scores compared to infants with a birth weight that is appropriate-for-gestational-age (AGA; Pallotto and Kilbride, 2006). Apart from IUGR, stunting can be caused by a nutritional deficit (such as protein-energy malnutrition) during the rapid growth of young children. Most often intrauterine malnutrition is followed by poor postnatal nutrition, and the combined and persistent effect of malnutrition across both periods results in seriously stunted growth (Dewey and Begum, 2011). Indeed, evidence from developing countries shows that stunting in early childhood is associated with poorer cognitive development and academic performance in later childhood (Grantham-McGregor, 1995; Grantham-McGregor et al., 2007). A recent review concluded that even mild but persistent malnutrition in early life (i.e., during the first 2 years of life) negatively influences reasoning, visuospatial functions, IQ, language development, attention, learning, and academic achievement, while supplementation with food can improve cognitive performance (Laus, 2011). In an interesting study, researchers randomly assigned 425 preterm infants to a “standard nutrient” group (who received either breast milk or standard formula) and “high nutrient” group (who received a higher protein-energy and micronutrient diet). The cognitive development of the children was then measured at 7½–8 years of age, and it was found that IQ (measured on the WISC) was higher in the high nutrient group, especially with respect to verbal IQ in boys (Lucas et al., 1998). A subgroup of these children (*n* = 76) was assessed again at 16 years, and a persistent effect of the high nutrient diet on verbal IQ was demonstrated. At this stage, brain MRIs were also undertaken and showed a larger volume of the caudate (which was correlated with higher verbal IQ), but only in males (Isaacs et al., 2008, 2009).

Obesity is a special form of malnutrition (overnutrition, as opposed to undernutrition which has been previously considered here), because the diet is likely to have low nutrient-density in conjunction with a high fat and carbohydrate content (Tanumihardjo et al., 2007). Obesity is of growing concern worldwide, with the number of overweight and obese children dramatically increasing over the past two decades. It was estimated in 2010 that 43 million children worldwide (including 35 million from developing countries) were overweight or obese, and this number is expected to continue to grow to 60 million by 2020 (De Onis et al., 2010).

Animal studies suggest that there may be a biological link between obesity and impaired cognitive performance that is related to insulin resistance and altered glucose metabolism (Jurdak et al., 2008). Furthermore, when rats were fed a high fat diet, it decreased neurogenesis in the hippocampus (Lindqvist et al., 2006). In addition, a maternal high-fat diet in mice altered the development of hippocampus in the foetus (Niculescu and Lupu, 2009), which may mediated by a decrease in the level of brain-derived neurotrophic factor (Molteni et al., 2002). A recent literature review concluded that overweight and obesity may result in poorer academic performance (measured as literacy, numeracy, and school grades; Burkhalter and Hillman, 2011), but only a few studies have researched a possible connection between obesity/overweight and cognitive performance. Li et al. (2008) described an association in 8–16-year-old children and adolescents between increased body mass index (BMI) and reduced cognitive performance, specifically visuospatial functioning as measured on the block-design test (a subtest of the WISC), but not attention, working memory (digit-span subtest) and academic performance (Wide Range Achievement Test). This association remained after adjusting for a range of covariates and potential obesity mediating factors. Palermo and Dowd (2012) did not find a similar association between increased body weight and cognitive performance as measured by the Woodcock Johnson Revised Test of Achievement and the Memory for Digit Span test. Bisset et al. (2012) examined weight status trajectories in 4–7-year-old children; overweight was not associated with cognitive outcomes as measured on the Kaufman's Assessment Battery for Children. The inconsistent findings in humans between obesity and cognitive development may be the result of the complexity of factors underlying these outcomes (Li et al., 2008). Moreover, associations between obesity and cognition that have been reported may be mediated by sociodemographic factors that include discrimination and isolation rather than through biological mechanisms (Palermo and Dowd, 2012).

#### Breakfast

The level of glucose metabolism in children's brains increases from birth until 4 years of age, reaching twice that of the adults' metabolic rate. This rate of glucose metabolism in children remains elevated until 9–10 years of age, before it declines to the adult level by late adolescence (Chugani, 1998). Therefore, regular meals and continuous glucose supply (to provide the brain with the required glucose for its high metabolism) is more important in children than in adults (Bellisle, 2004). Accordingly, children are more prone to the adverse effect of overnight fasting, and breakfast is a very important meal to provide fuel to the brain in the morning (Bellisle, 2004). A systematic review concluded that having breakfast is beneficial for cognitive function and development, especially in malnourished children. A lack of studies conducted into the optimal breakfast including type, composition and portion size exists but (Hoyland, 2009) carbohydrate rich, low-glycaemic food for breakfast that provides a continuous supply of glucose is known to facilitate better cognitive performance (Bellisle, 2004; Ingwersen et al., 2007; Micha et al., 2011).

A recent study showed that the brain's gray and white matter volume differed in various parts of healthy children's brain, according to the type of breakfast they ate (Taki et al., 2010). The researchers suggested that the difference may be due to the different glycemic index of the different breakfast staple types. The authors also proposed that if different breakfast types affect gray and white matter volume in the brain, they may in turn influence cognitive function. Therefore, the type of breakfast children eat can potentially have a long-term influence on cognitive development (Taki et al., 2010).

#### Dietary pattern and diet quality

Since individuals consume combinations of foods (which may contain other bioactive compounds that could act synergistically or antagonistically within or between food groups; Tangney and Scarmeas, 2011), it is important to investigate diet as a broadly encompassing variable in association with cognitive outcomes. Furthermore, as Tangney and Scarmeas (2011) state, if research shows an association between diet (as comprehensively measured) and better health outcomes, it may be easier to implement changes in terms of dietary interventions.

Some researchers have investigated the influences of overall diet on neurocognitive development during childhood. Gale et al. (2009) considered dietary patterns in infancy in relation to cognitive development and found higher full-scale IQ (measured on the Wechsler Preschool and Primary Scale of Intelligence test) at 4 years of age in children who consumed higher amounts of fruit, vegetables and food prepared at home during infancy (i.e., between 6 and 12 months). The association remained significant after adjusting for a wide range of factors, including socioeconomic status, maternal IQ and education. A cross-sectional study reported by Theodore et al. (2009) examined the association between (i) the intake of specific food groups in 3½ years old children and in the same children again at 7 years of age and (ii) their cognitive development measuring on the Stanford–Binet Intelligence Scale (at 3 years) and on the WISC-III (at 7 years). These researchers found that a higher level of consumption of fish at 7 years of age and bread and cereals at 3½ years of age was associated with higher IQ scores, whereas those children at the age of 3½ who consumed margarine every day scored significantly lower on IQ. Northstone et al. (2011) reported that higher scores on the “health conscious” dietary pattern (which included more salad, rice, pasta, and fruits) at 3 years of age were associated with higher IQ score on the WISC-III when these same children were tested aged 8½ years, compared to those children on the “processed” dietary pattern (with high fat and sugar content), after adjusting for a wide variety of potential confounders. In the same study, Smithers et al. (2012) examined six different dietary patterns and found negative associations between (i) the “discretionary pattern” (which contains biscuits, sweets, soft drinks, and snacks) at 6, 15, and 24 months of age, and ready-prepared baby foods at 6 and 15 months of age and (ii) IQ scores at 8 years of age (measured on the WISC). Smithers et al. (2012) also reported positive associations between children's IQ at age 8 years and “breastfeeding pattern” (measured at 6 months), “home-made contemporary” (legumes, fruits, fruit juices, cheese, egg) at 15 and 24 months, “home-made traditional” (vegetables, meat, sauces) at 6 months (but not at 15 and 24 months), and “ready-to-eat” food pattern (biscuits, breads, cereals, yoghurt) at 24 months.

## Conclusion and recommendations for future research

The majority of studies, which have investigated the association between nutrition and cognitive development, have focused on individual micronutrients, including omega-3 fatty acids, vitamin B12, folic acid, zinc, iron, and iodine. The evidence is more consistent from observational studies, which suggest these micronutrients play an important role in the cognitive development of children. However, the results from intervention trials of single nutrients are inconsistent and inconclusive, prompting the need for better controlled and more adequately powered studies in the future. It is plausible that children living in poor countries may encounter more multiple micronutrient deficiencies, as opposed to children living in rich countries who are reasonably well nourished (and where a small deficiency in one nutrient may not result in measurable, long-term change in cognitive outcomes, due to compensation over time). These are important considerations, because nutrients do not act alone; rather, they have in some contexts synergistic and in other contexts antagonistic effects with each other.

Individuals consume combinations of food and poor overall diet can cause multiple macro-and micronutrient deficiencies and imbalances. If an overall healthy diet synergistically enhances cognitive development in children, then public health interventions should focus on the promotion of overall diet quality rather than isolated micronutrients or dietary components consumed by children and adolescents.

### Conflict of interest statement

The authors declare that the research was conducted in the absence of any commercial or financial relationships that could be construed as a potential conflict of interest.
